# Prebiotic Chemistry: The Role of Trimetaphosphate in Prebiotic Chemical Evolution

**DOI:** 10.3389/fchem.2022.941228

**Published:** 2022-07-13

**Authors:** Dingwei Gan, Jianxi Ying, Yufen Zhao

**Affiliations:** ^1^ Institute of Drug Discovery Technology, Ningbo University, Ningbo, China; ^2^ Qian Xuesen Collaborative Research Center of Astrochemistry and Space Life Sciences, Ningbo University, Ningbo, China; ^3^ Department of Chemical Biology, College of Chemistry and Chemical Engineering, Xiamen University, Xiamen, China

**Keywords:** trimetaphosphate, prebiotic chemistry, peptide, nucleotide, origin of life

## Abstract

Life’s origins have always been a scientific puzzle. Understanding the production of biomolecules is crucial for understanding the evolution of life on Earth. Numerous studies on trimetaphosphate have been conducted in the field of prebiotic chemistry. However, its role in prebiotic chemistry has been documented infrequently in the review literature. The goal of this thesis is to review the role of trimetaphosphate in the early Earth’s biomolecule synthesis and phosphorylation. Additionally, various trimetaphosphate-mediated reaction pathways are discussed, as well as the role of trimetaphosphate in prebiotic chemistry. Finally, in our opinion, interactions between biomolecules should be considered in prebiotic synthesis scenarios since this may result in some advances in subsequent research on this subject. The research establishes an essential and opportune foundation for an in-depth examination of the “mystery of life".

## Introduction

The question “how did life emerge on the early earth” has perplexed scientists for decades. Scientists have recently made a concerted attempt to accomplish this. Charles Darwin said, “Phosphorus played an irreversible role in the creation of life, and phosphorus is the main source of life on Earth” (Zimmer, 2009). F H Westheimer pointed out that for genetic material like DNA to exist, a connecting link that is at least divalent must be present as well. The connecting unit should have a third, ionizable group to ensure that the final substance retains its membrane charge. Thus, phosphorus is an irreplaceable role (Westheimer, 1987). It can be said that phosphorus plays an absolutely important role in all life forms, and this chemical is widely involved in metabolism and biochemical reactions (Karki et al., 2017).

However, the availability of phosphates in prebiotic chemistry has been questioned. According to early geological research, most of the phosphorus on the primitive earth was insoluble in water and existed in the form of apatite (Gulick, 1957; Keefe and Miller, 1995; Macia et al., 1997; Ulian et al., 2021). As a result, a common perception is that phosphate was strongly limited in prebiotic chemical development, and thus no such indispensable element in prebiotic processes, unless it is derived from meteorites, lightning and sources (de Graaf et al., 1995; Ritson et al., 2020). Burcar, Bradley, et al. proposed that the formation of a urea/ammonium formate/water (UAFW) eutectic solution could promote phosphorylation, which results in increased sources of phosphate with different solubilities (Burcar et al., 2016). Polyphosphates, in contrast to orthophosphates, are long-chain molecules generated when orthophosphates are joined together via stable P-O-P bonds during dehydration (Brown and Kornberg, 2004). Polyphosphates have a higher phosphorylation capability. Thus, polyphosphates are required for bioactivation and phosphorylation, and they are also more active and soluble inherently than orthophosphates (Omelon et al., 2009; Dürr-Mayer et al., 2021). Numerous researchers have attempted to convert orthophosphates to polyphosphates in water using a variety of condensing agents (Weber, 1981, 1982; Hagan et al., 2007). Griffith et al. discovered that iron (III) phosphate may be reduced to iron (II) phosphate and carbon dioxide in the presence of carbon monoxide and that iron (III) phosphate can form pyrophosphate in the presence of hydrogen sulfide. At somewhat increased temperatures, pyrophosphate can then spontaneously polymerize into linear and cyclic polyphosphates (Griffith et al., 1977). Under prebiotic conditions, this cascade of processes was feasible. Yamagata et al. conducted experiments replicating the conditions of volcanic magma and studied volatile condensates in volcanic gases. Additionally, studies revealed that volcanic activity can result in the formation of water-soluble polyphosphates, such as trimetaphosphate (P_3_m) (Yamagata et al., 1991). Pasek et al. also proposed that the P_3_m could be generated among other polyphosphates (Pasek et al., 2008), Gibard et al. demonstrated that diamidophosphate could form through the reaction of NH_4_^+^ with the meteorite mineral schreibersite. Thus the N-P compounds could generate P_3_m (Gibard et al., 2018). Then after, Gibard et al. also showed that diamidophosphate could form through the reaction of NH_4_^+^ with the meteorite mineral schreibersite. Thus the N-P compounds could generate P_3_m (Gibard et al., 2019). Additionally, Britvin et al. reported the cyclic tetraphosphate from mineral. (Britvin et al., 2021). The researchers identified pyrophosphate and triphosphate in the volcanic jet condensate from Wusu Mountain (Schwartz, 2006). All of the above evidences infer the existence of polyphosphates before the existence of living matter, and promotes the occurrence of chemical reactions and the evolution of life. Furthermore, numerous investigations into the phosphorylation and polymerization of biomolecules and polyphosphates have been conducted in this area (Ponnamperuma et al., 1963b; Schwartz and Fox, 1964; Ponnamperuma and Mack, 1965; Ferris, 1968; Fox and Waehneldt, 1968; Lohrmann and Orgel, 1968; Rabinowitz et al., 1968; Schwartz and Ponnamperuma, 1968; Rabinowitz et al., 1969; Rabinowitz, 1970; Lohrmann and Orgel, 1971; Handschuh et al., 1973; Lohrmann, 1975; Hulshof and Ponnamperuma, 1976; Schoffstall, 1976; Yamagata et al., 1979; Yamanaka et al., 1988; Schwartz, 2006; Holm and Baltscheffsky, 2011; Kee and Monnard, 2017; Christ et al., 2020; Serov et al., 2020; Brunk and Marshall, 2021; Glonek, 2021; Ying et al., 2021).

The purpose of this paper is to review the prebiotic synthesis and phosphorylation of several biomolecules with the participation of P_3_m, such as peptides, nucleotides, oligonucleotides etc. These prebiotic investigations of biomolecules not only have essential guiding value for the development of the life sciences, but also underscore the important role of P_3_m in the evolution of prebiotic chemistry.

## Prebiotic Peptide Synthesis Involved by P_3_M

**FIGURE 1:**
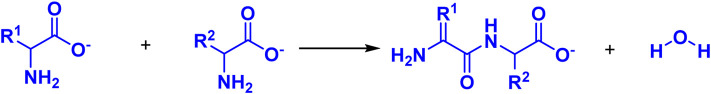
Reaction for the condensation of amino acids in water to dipeptides.

**FIGURE 2:**
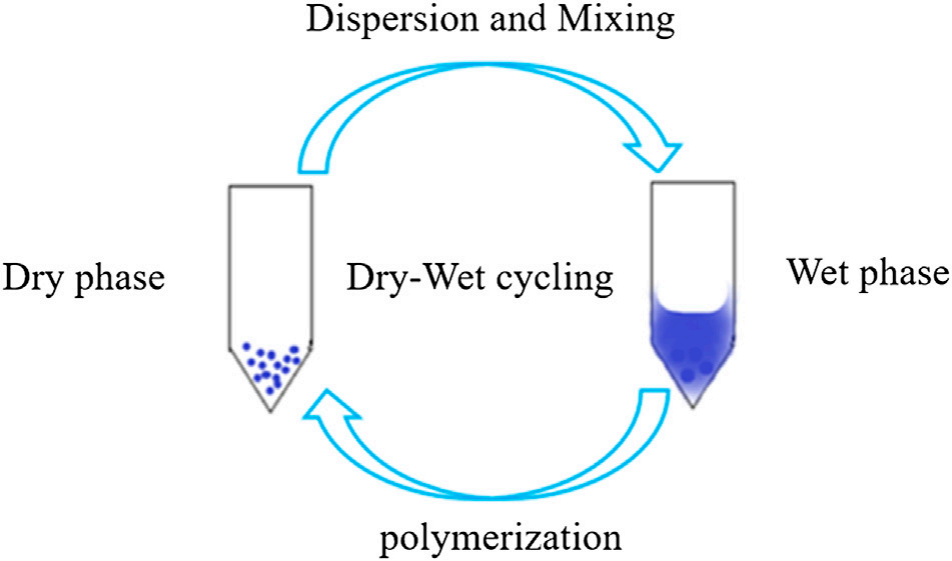
Wet and dry cycle diagram.

The synthesis of prebiotic peptides that occurred under early Earth conditions has long been the focus of the field of the origin of life (Urey, 1952; Leach et al., 2006; Fiore, 2019; Micca Longo et al., 2021). Due to the fact that the synthesis of peptides from amino acids requires energy input and the removal of water molecules (Figure 1), the reaction is often not favored in aqueous solutions, which is also thermodynamically unfavorable for the reaction to occur. As a result, early research on prebiotic peptides was conducted in dry settings (Hayakawa et al., 1967; Yanagawa et al., 1990). Wet-dry cycling is viewed as a mechanism for driving the condensation reaction that generates biopolymers, and numerous publications have described the synthesis of prebiotic peptides under dry-wet cycling circumstances (Figure 2) (Forsythe et al., 2015; Campbell et al., 2019). Additionally, the majority of the early primitive earth environments were aqueous, and adding certain activators to the aqueous environment or altering the reaction temperature, pH, and other conditions in order to make the reaction thermodynamically favorable may be consistent with the emergence of prebiotic peptides. Numerous indications for the condensate’s prebiotic chemistry contribution. For example, urea can help phosphorylate nucleosides and glycerol (Knecht et al., 2002). Certain minerals and clays also have the potential to catalyze the process of prebiotic source phosphorylation, and many minerals are positively charged and can adsorb negatively charged phosphates and phosphate esters, providing a relatively ideal polymerization environment for phosphorus compounds. A study conducted by Gull et al. (2014) demonstrated that inorganic phosphates adsorbed on silica can be condensed at relatively low temperatures (Gull et al., 2014).

P_3_m appears to stimulate the synthesis of prebiotic-derived peptides under a range of circumstances (Rabinowitz, 1970; Yamanaka et al., 1988; Yamagata and Inomata, 1997; A and E, 2002; Gu et al., 2011; Sibilska et al., 2017; Sibilska et al., 2018; Ying et al., 2018a; Ying et al., 2018b). Numerous variables influence the yield of prebiotic dipeptides, including temperature, pH, reaction system, wet and dry environment, and so on. Although we do not know precisely how prebiotic sources evolved on early Earth, we can alter these reaction conditions and hence examine putative evolutionary pathways for life.

**FIGURE 3:**
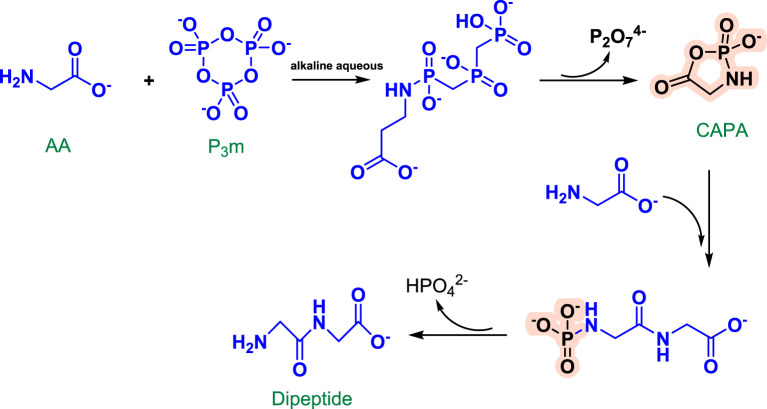
Reaction mechanism of Gly_2_ formation from P_3_m in alkaline solution [redrawn from (Chung et al., 1971)].

Rabinowitz et al. initially described the condensation reaction of glycine (Gly) and P_3_m in 1961. Following that, Chung et al. developed a reaction mechanism for the synthesis of Gly_2_ in an alkaline solution using Gly and P_3_m (Chung et al., 1971). The amino group of Gly likely attacks P_3_m first, forming the open-chain molecule Gly-N-triphosphate. Following intramolecular condensation, a cyclic acylphosphoramidate is formed as an active intermediate (CAPA). CAPA is then attacked by another Gly to produce Gly_2_-N-phosphate, which is quickly hydrolyzed to generate Gly_2_ (Figure 3). Yamanaka et al. added phosphate and Gly in equimolar quantities to an aqueous solution with a pH of 4.0–9.0 and a temperature of 38°C. The reaction solution was examined using high-performance liquid chromatography and the ninhydrin reaction method to detect the Gly tetramer and hexamer. They discovered that the yield was greatest at a pH of about 7. Additionally, yields varied with different phosphates, and the reaction with P_3_m was approximately tenfold that of tetrametaphosphate under the identical circumstances (Yamanaka et al., 1988). This emphasizes the critical role of P_3_m in the evolution of life. Yamanaka and colleagues postulated that the condensation of amino acids with P_3_m may occur under slightly acidic settings; subsequent analysis confirmed that the condensation of oligoglycines with P_3_m occurs most efficiently under neutral or weakly acidic conditions. Condensation occurs differently in aqueous solution than it does with Gly. Gly_2_-N-triphosphate is rarely formed in neutral or acidic circumstances (Yamanaka et al., 1988).

Along with synthesizing peptides in alkaline and acidic conditions, Izabela Sibilska et al. did comparative tests on glycine and alanine in the presence or absence of P_3_m at temperatures ranging from 0 to 100°C and pH values ranging from 1 to 12. The data indicate that glycine and alanine rapidly produce peptides in the absence of harsh near-boiling temperatures, excessive pH values, or dry solid residues at P_3_m. In comparison, peptides are synthesized in the presence of P_3_m under a broader range of circumstances, including ambient temperature, neutral pH, and water (Sibilska et al., 2018). The above indicates that the synthesis of prebiotic peptides is promoted by P_3_m in acidic, alkaline, or neutral aqueous solutions.

Additionally, similar to some of the conditions outlined previously, P_3_m can facilitate or accelerate the production of prebiotic-derived peptides. We have discovered that P_3_m could also act as a catalyst for the synthesis of cyclic dipeptides in an aqueous solution (Ying et al., 2018b; Guo et al., 2021). In addition to the parameters outlined above, there may be a broader range of circumstances for the synthesis of prebiotic peptides in the primordial Earth environment, but we are unable to recreate the prebiotic period’s environment to undertake relevant investigations. This is a perplexing subject in the field of life origins. Through continual investigation, it is possible for us to identify some chemical beginnings of life and to reproduce as many prebiotic-era processes as possible.

## Nucleoside Prebiotic Phosphorylation Mediated by P_3_M

Nucleotides are the fundamental building units of RNA and DNA, the two polymers responsible for life’s biochemistry (Azzam and Liu, 2013; Shivakumar et al., 2019; Miggiano et al., 2020; Cai et al., 2021). The investigation of their synthesis has made a substantial contribution to our understanding of chemical evolution and the chemical origins of life. Thus, from the perspective of the origin of life, how did nucleotides assemble from their various chemical components (ribose, adenine, guanine, cytosine, and uracil) or their respective constituent small molecules (e.g., HCN, HCHO, and phosphate)?

Under replicated pristine Earth circumstances, the synthesis of the bases adenine and guanine, as well as the sugars ribose and deoxyribose, has been demonstrated (Oro, 1963). Monosaccharides have been synthesized by Gabel and Ponnamperuma in 1967 by formaldehyde; purines and pyrimidines have also been shown to be formed by prebiotic reactions by Oro and Kimball and Ponnamperuma et al. (Ponnamperuma and Mack, 1965; Gabel and Ponnamperuma, 1967). Additionally, the nucleobases in meteorites was also reported by Burton et al. (Burton et al., 2012). The process of nucleotide synthesis involves the removal of water molecules: Purines or pyrimidines first react with ribose or deoxyribose to generate nucleosides, which are then phosphorylated to form nucleotides. When several nucleotides are polymerized, an ester link is created between the nucleotide’s phosphate residue and the pentose residue’s hydroxyl group (Mizuno et al., 1975). It is worth mentioning that the preceding series of chemical evolutionary processes result in the formation of water molecules, making such reactions thermodynamically unfavorable in water. Thus, in what kind of environment did such chemical evolution occur during the early Earth’s history? Additionally, the supply of phosphorus during chemical evolution is a point of discussion.

**FIGURE 4:**
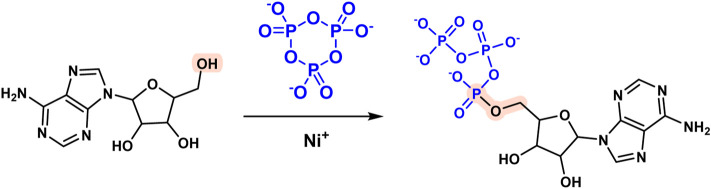
Reaction of nucleoside 5′-triphosphate formation mediated by P_3_m.

Phosphorus is required for the formation of prebiotic sources of biological macromolecules like RNA and proteins and phosphorylation of nucleosides is a critical link in RNA synthesis (Lin et al., 2011; Pasek et al., 2013; Altwegg et al., 2016; Martínez-Bachs and Rimola, 2019; Toparlak and Mansy, 2019; Liu et al., 2021). The first reagent used for phosphorylation of nucleoside prebiotic sources was a mixture of urea and inorganic phosphate. One important example is nucleoside 5′-triphosphate, a precursor of RNA in modern biology. Kim HyoJoong et al. synthesized large amounts (2-3 percent) of nucleoside 5′-triphosphate by heating nickel (II) by evaporation in the presence of borate, urea, salt, and P_3_m (Figure 4) (Kim and Benner, 2021). Additionally, phosphorus-containing minerals have been implicated in chemical evolution as a source of phosphorus (Pasek and Lauretta, 2005; Saladino et al., 2006; Saladino et al., 2009; Gull et al., 2015; Burcar et al., 2016; Toner and Catling, 2020).

Human cells have a composition similar to that of seawater in modern biological systems (Kuro, 2021). This fact further suggests that the ocean may have been a reaction medium prior to the origin of life (Zhang and Kim, 2010; Abida et al., 2013). Scientists have done some significant experiments in recent years. In aqueous solutions, the dehydration condensation process was shown to be achievable when paired with the hydrolysis of specific condensates. As a prebiotic condensation agent, P_3_m plays a critical role in the prebiotic phosphorylation of nucleosides (Schramm et al., 1961; Ponnamperuma et al., 1963a; Ponnamperuma et al., 1963b; Kimball and Oro, 1971; Fuller et al., 1972; Schoffstall, 1976). Specially, Moretti and Muller proposed that RNA oligomers could be constructed by P_3_m (Moretti and Müller, 2014), and the work by Akoopie et al. and Dolan et al. allow the buildup beyond just dimers (Dolan et al., 2015; Akoopie et al., 2021).

**FIGURE 5:**
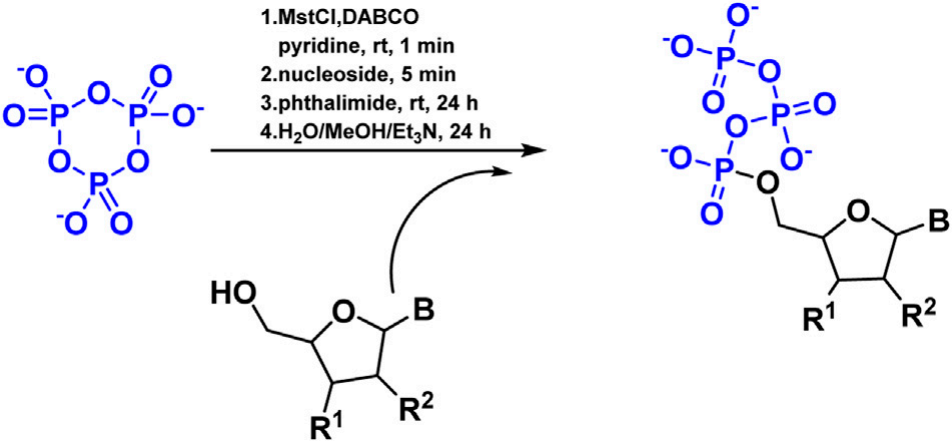
Reaction of NTPs formation with P_3_m in the presence of DABCO.

Since the 1960s, chemists have attempted to triphosphorylate nucleosides and other alcohols using P_3_m. However, it appears as though this route has been abandoned due to low yields. The first successful synthesis of nucleoside triphosphates (NTPs) was described, utilizing P_3_m as a critical reagent (Figure 5). This was performed by reacting the tetrabutylammonium salt of P_3_m in pyridine with mesitylenesulfonyl chloride in the presence of DABCO, followed by the addition of properly protected nucleosides and phthalimide (Samy et al., 2016).

**FIGURE 6:**
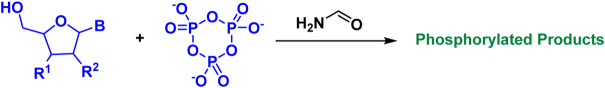
Reaction mechanism of dipeptide formation from P_3_m in acidic solution [redrawn from (Ying et al., 2021)].

Schoffstall proposed a prebiotic phosphorylation pathway for nucleosides in the presence of formamide that is mediated by P_3_m (Schoffstall, 1976; Schoffstall et al., 1982; Schoffstall and Mahone, 1988). Phosphorylation can occur at any point on the ribose throughout this process, finally resulting in the creation of cyclized nucleotides, nucleoside diphosphates, and nucleoside cyclic phosphates were synthesized in formamide solutions containing phosphate and deoxynucleoside at 70° and 120°. These events can be thought of as examples of a distinct sort of prebiotic phosphorylation known as non-aqueous phosphorylation (Figure 6).

Hyo-Joong Kim et al. suggested a prebiotic synthesis of phosphorylated nicotinamide ribose that conveniently provides the adenosine phosphate component of this and other RNA cofactors (Kim and Benner, 2018). The mechanisms of nucleoside triphosphorylation were crucial in the emerging “RNA world” because they provided high-energy substrates for reactions such as RNA polymerization. F. Chizzolini et al. established that P_3_m is suitable for use in mild prebiotics. Under appropriate conditions, it interacted with nucleosides to generate NTP, hence boosting RNA synthesis by T7 RNA polymerase and polymerase ribozymes (Chizzolini et al., 2021).

## P_3_M-Mediated Phosphorylation of Other Biomolecules

Phosphorylation is required for the biochemical functions of living organisms. As a result, the study of the genesis of life is primarily concerned with its origin. The early Earth most likely contained the majority of the components necessary for the emergence and development of life (Izabela et al., 2017). Phosphorylation happened not just on nucleosides, but also on other organic molecules such as amino acids, alcohols, etc.

Phosphorylation of amino acid is a well-characterized post-translational alteration of proteins involved in biological activities (Lai et al., 2017; Kumar and Thompson, 2019; Zientara-Rytter and Subramani, 2019). Phosphorylation, as a critical post-translational modification of proteins, plays a critical role in cell signaling, functional control, and energy transfer in modern organisms (Derouiche et al., 2012; Hunter, 2012; Petkowski et al., 2019; Hu et al., 2020). The primordial source of phosphorylated amino acids required the early Earth to have an adequate supply of amino acids and phosphorus-containing molecules. On early Earth, there were two primary suppliers of amino acids. They were referred to as the extrinsic and endogenous pathways, respectively. The extrinsic pathway refers to amino acid synthesis occurring outside of the earth and being delivered to the earth via interstellar dust particles, meteorites, and so on, whereas the endogenous pathway refers to the atmospheric mixture within the earth *via* a series of discharges. Abiotic reactions such as hydrothermal fountains synthesize amino acids (Miller, 1953; Zaia et al., 2008; Lin et al., 2020; Takeuchi et al., 2020).

**FIGURE 7:**
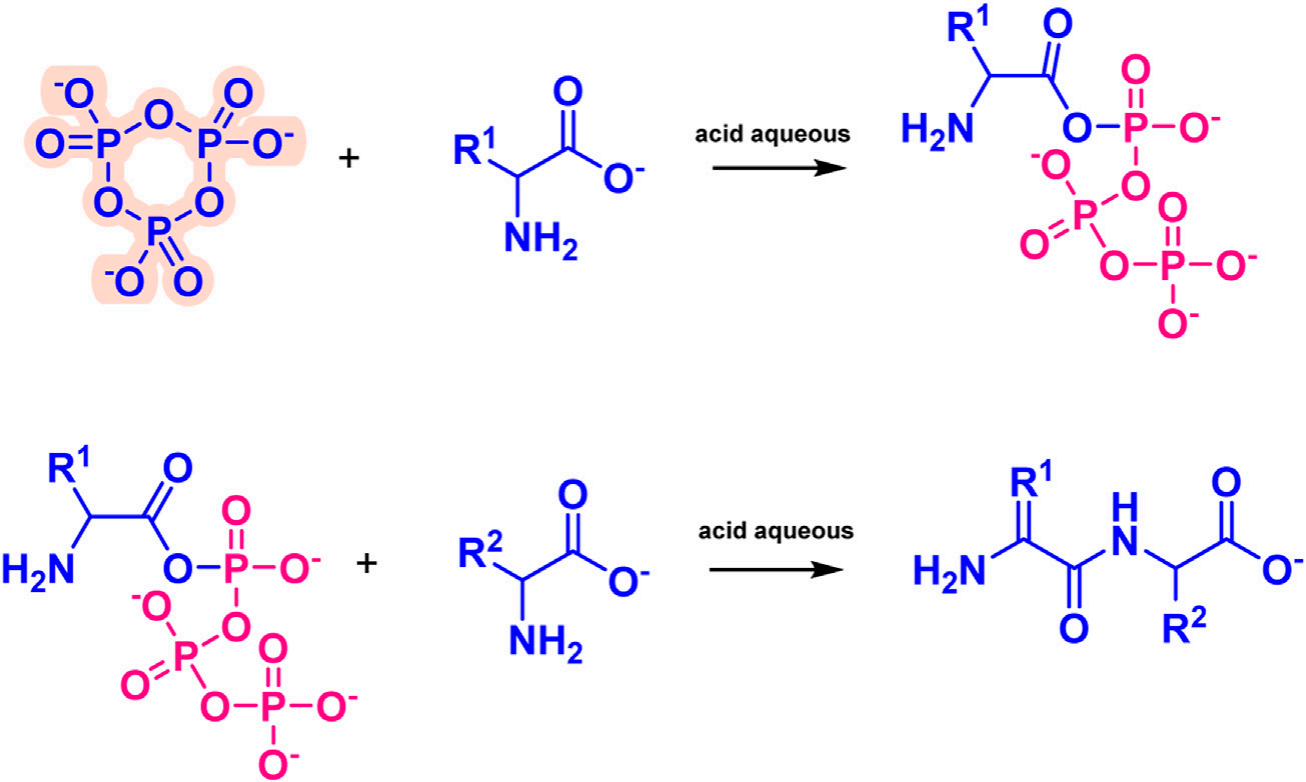
Reaction pathway for phosphate-catalyzed synthesis of nucleoside prebiotic sources in the presence of formamide.

**FIGURE 8:**
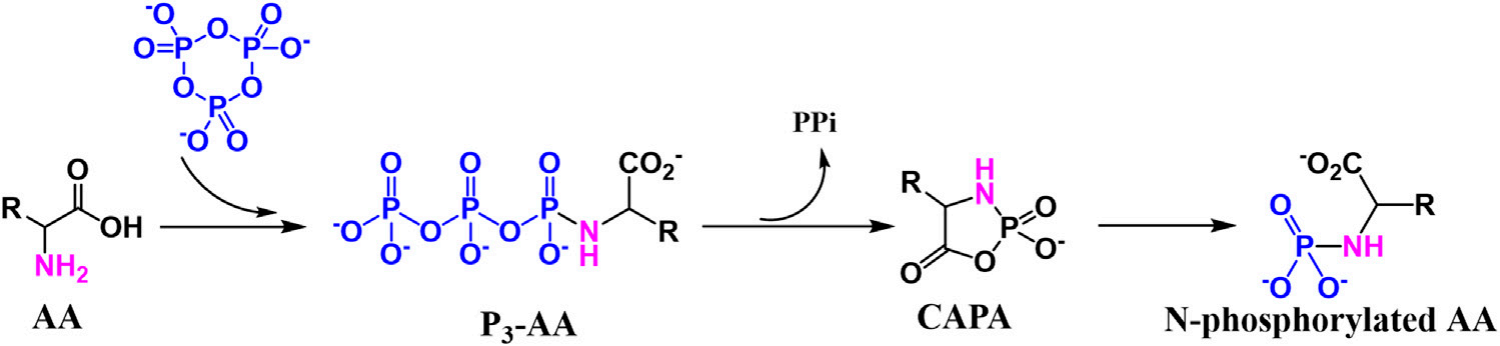
Formation mechanism of N-phosphorylated amino acids.

Amino acid phosphorylation is divided into oxygen phosphorylation (O-phosphorylation) and nitrogen phosphorylation (N-phosphorylation), and the two types of prebiotic amino acid phosphorylation mentioned above are described next. The O-phosphorylation is a process that requires the participation of ATP for modern organisms, which plays an indispensable role in the normal metabolism of living organisms. we have proposed a reaction which contained O-phosphorylated (Figure 7). The P_3_m can react with amino acid in acid aqueous environment, which results the O-phosphorylation (Hill and Orgelr, 2002). The N-phosphoryl amino acid is the simplest phosphoryl amino acid without an ester group on phosphorus, and its possible formation pathways include the reaction of amino acid with polyphosphate, especially the reaction of amino acid and P_3_m (Rabinowitz et al., 1969; Feng et al., 2009). The main formation mechanism is as follows (Figure 8), the amino group of the α-amino acid attacks P_3_m to form the P_3_-AA intermediate, which subsequently becomes cyclic acyl phosphoramidite (CAPA) and pyrophosphate. Since CAPA is unstable, it is subsequently hydrolyzed by ring-opening to N-phosphoryl amino acids (Feng et al., 2010).

**FIGURE 9:**
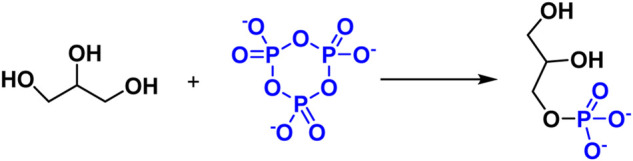
Reaction of Glycerol phosphorylation with P_3_m.

Glycerol and glycerophosphate have been shown to be potential prebiotic molecules, Maheen Gull et al. investigate the phosphorylation of glycerol using a variety of inorganic phosphates, including sodium phosphate, P_3_m, and struvite. P_3_m was discovered to have a favorable influence on the probiotic phosphorylation of glycerol, and non-aqueous solvents were found to be advantageous for the prebiotic synthesis of biomolecules (Figure 9) (Gull et al., 2017).

**FIGURE 10:**
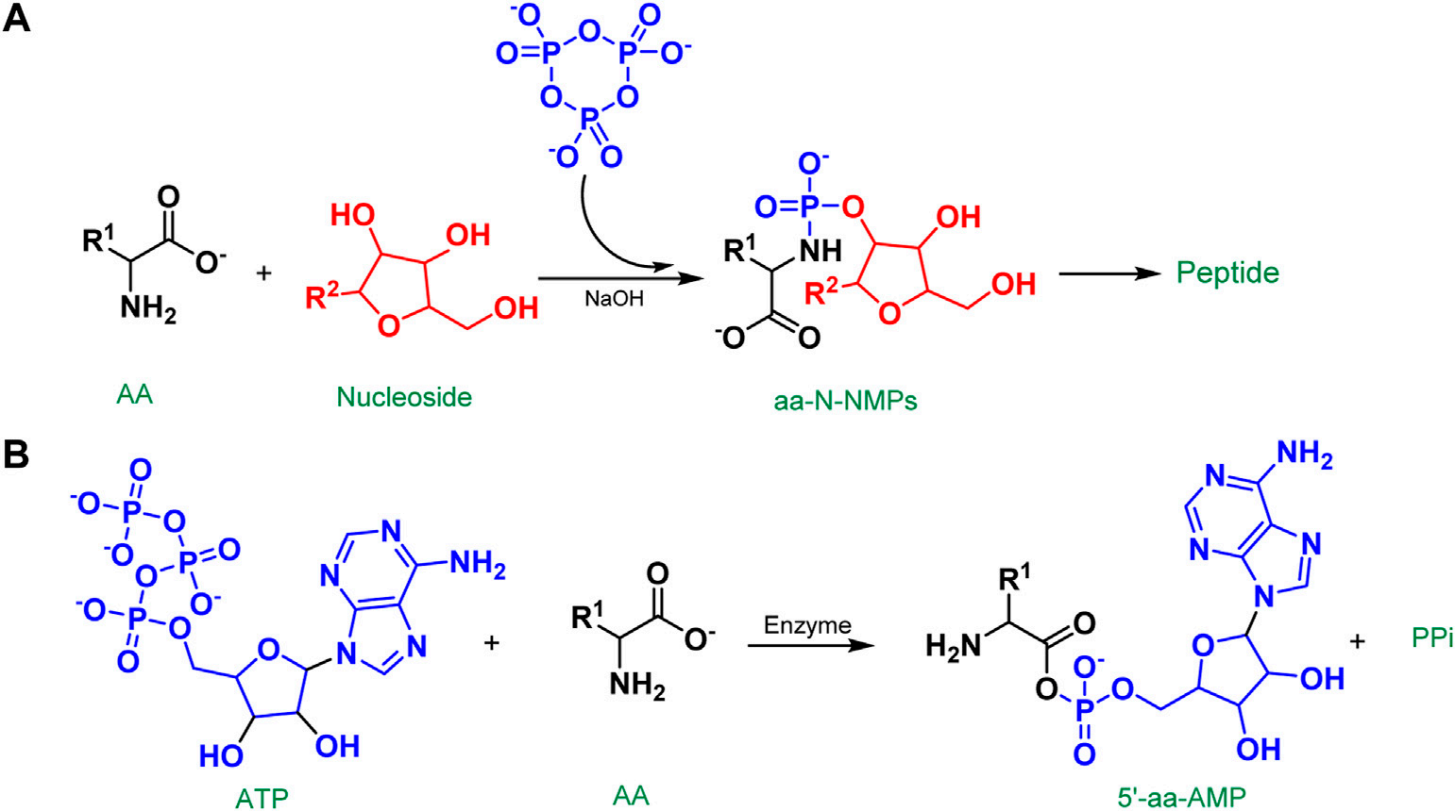
**(A)** The chemical model of the co-origin of codons and proteins; **(B)** Formation of the 5′-aa-AMP in organism.

We have stressed the function of P_3_m in fostering chemical evolution in their chemical model of codon and protein co-origin (Ying et al., 2018a). Through a reaction with amino acids and nucleosides in an alkaline aqueous solution, the P_3_m generates a critical intermediate molecule, nucleotide amidate (aa-*N*-NMP). The aa-*N*-NMP is a molecule that joins amino acids and nucleosides via a phosphate group. As the nucleosides and amino acids in the reaction vary, the structure of the resulting aa-*N*-NMP changes as well, changing the energy required to activate it. As a result, the final dipeptide yields vary (Figure 10A). As we know, aminoacyl-tRNA (aa-tRNA) is generated by transferring an aminoacyl group from 5′-aminoacyladenylates (5′-aa-AMPs) to the tRNA in modern biochemistry. Wonderfully, the structure of aa-*N*-NMP is comparable to that of 5′-aa-AMPs, a critical activation intermediate in modern organism peptide synthesis. 5′-aa-AMPs are synthesized in living organisms through the interaction of ATP with amino acids, which is catalyzed by a variety of enzymes and cofactors (Figure 10B). Thus, the aa-*N*-NMP as a mediator of prebiotic tRNA to assist in the activation of amino acids to form peptides could be a potential pathway by which aminoacyl groups from aa-*N*-NMP are transferred to plausible prebiotic tRNAs. So, in early earth conditions, with environmental changes and other considerations, did 5′-aa-AMP evolve step by step from aa-*N*-NMPs as an intermediate?

## Conclusion and Outlook

The beginning of life is an intriguing and perplexing issue. P_3_m, as a significant condensation agent in prebiotic chemistry, exhibits superior condensation ability and higher water solubility in comparison to other condensation agents, bolstering the theory that life started from the ocean, a shallow pool or other source (Corliss et al., 1979; Kelley et al., 2001; Martin et al., 2008; Burcar et al., 2016). Numerous studies on its involvement in the genesis of life have been conducted in recent years, and P_3_m is steadily demonstrating its particular appeal in the field of prebiotic chemistry, aiding us in unraveling the riddle of life step by step.

It is unquestionably necessary to investigate non-biosynthetic peptides or nucleotides alone to replicate the early Earth environment in the study of prebiotic chemistry, and scientists have established a certain research base in this area. It is conceivable that chemically selective peptides-nucleotides interactions were involved in the processes of chemical evolution that have contributed to the origin of the genetic code. The shared origin of the genetic code and proteins adds another dimension to the field. While we cannot return to the early Earth to see the entire picture, we can go further and deeper into prebiotic scenarios to identify possible forms of life on the early Earth.
